# Supplementary material for: Protective role of renal proximal tubular alpha-synuclein in the pathogenesis of kidney fibrosis
Source: Nat Commun. 2020 Apr 23;11:1943. doi: 10.1038/s41467-020-15732-9 (PMC7181766; doi:10.1038/s41467-020-15732-9)
Supplement: Supplementary file 1 — Supplementary Information [file 41467_2020_15732_MOESM1_ESM.pdf]

SUPPLEMENTARY INFORMATION

Protective role of renal proximal tubular alpha-synuclein in the pathogenesis of kidney fibrosis

Bozic *et al.*

SUPPLEMENTARY METHODS

Genotyping of SNCA and PEPCK-Cre by PCR

PEPCK-Cre transgenic mice were identified using a pair of Cre-specific primers, 5´-CGGTGCTAACCAGCGTTTTC-3´ and 5´-TGGGCGGCATGGTGCAAGTT-3´. PEPCK-Cre primers generated a 465-bp fragment. SNCA mutant and WT band were identified using a $SNCA^{loxP}$ specific primers 5´-AAGAGCTAGTGGTGGGCAGA-3´ and 5´-GCTGGGCACAGTGTTGATTG-3´. Flox primers amplified a 440-bp fragment for the SNCA floxed allele and a 390-bp fragment for the SNCA WT allele.

Adenine-induced renal tubulointerstitial fibrosis in mice

Male $PEPCK^{Cre+}$ $SNCA^{wt/wt}$ and $PEPCK^{Cre+}$ $SNCA^{flox/flox}$ mice (8-10 weeks old) were fed a standard pellet chow or an adenine rich diet (0.25% Adenine) during 4 weeks (Ssniff, Germany)[1, 2]. Body weight and BUN levels was recorded weekly. Following the completion of the experiment, blood was collected by cardiac puncture and the animals were perfused with PBS through a puncture in the left ventricle. One part of the kidney was fixed in 4% paraformaldehyde/PBS for histologic examinations after embedding in paraffin and/or Bright Cryo-M-Bed compound (Bright Instrument Co). The remaining kidney tissue was snap-frozen in liquid nitrogen and kept at -80°C for protein and mRNA extractions.

Quantitative real-time PCR

Gene-specific TaqMan probes for real-time PCR used in this study are as follows: human α-synuclein (Hs01103383_m1), GAPDH (Hs99999905_m1), mouse α-synuclein (Mm01188700_m1), vimentin (Mm00449201_m1), α-SMA (Mm01546133_m1), collagen I (Mm00483888_m1), TGF-β1 (Mm00441724_m1), fibronectin (Mm01256734_m1), TBP

(Mm00446971_m1) (all from Applied Biosystems-Life Technologies S.A., Madrid, Spain), and mouse cadherin-16 (Mm.PT.58.31371125) (IDT Integrated DNA Technologies, USA).

Cross-linking and immunoprecipitation

HEK293T cells (ATCC® CRL-3216™) were co-transfected with SNCA-flag and HA-p38 PCNA plasmids using lipofectamine (ThermoFisher), according to the manufacturer´s instructions. HA-p38 PCNA was a kind gift from Dr. Guadalupe Sabio (CNIC, Madrid, Spain). SNCA-flag plasmid was constructed by amplifying human alpha-synuclen (450 bp) by PCR from SIN-PGK-hsynuclein-WHV plasmid and subcloning it into FLAG3´ pcDNA3 (HindIII and Xbal). After transfection, cells were washed once with PBS and collected in PBS with Complete Protease Inhibitor Mixture, EDTA-free (Roche). Immediately before use, the crosslinker dithiobis(succinimidyl) propionate (DSP, A35393, ThermoScientific) was prepared at 33x in DMSO. Samples were crosslinked with 650 µM DSP for 30 min at 37°C with agitation. The reaction was quenched with the addition of 2 M Tris, pH 7.5, to 25 mM final concentration and incubated for 15 min at room temperature (RT). Samples were sonicated on ice, and Triton X100 was added to a final concentration of 1% and incubated 10 min at 4°C. Lysates were centrifuged at 16.000 g 30 min at 4ºC and supernatant was incubated with 40 µL of anti-HA agarose beads (26181, Pierce) for 2 hours at 4°C to immunoprecipitate p38-HA. Beads were washed and eluted in loading buffer. To examine the interaction of p38-HA with SNCA-flag, immune complexes were analyzed on Western blots.

Western blot analysis

HK-2 cells were treated as indicated and harvested by two washes with ice cold PBS. They were subsequently lysed with lysis buffer (50 mM Tris (pH 7.5), 150 mM NaCl, 1% Triton X-100, 0.5 µM EDTA, 1 mM Na_3VO_4, 2 mM PMSF and protease inhibitor cocktail) and rotated at 4°C for 1 hour. After rotation, cell lysates were centrifuged at 13.000 rpm for 15 min, 4°C and the supernatant was saved at -80°C. For the analysis of phosphorylated proteins, cells were lysed with lysis buffer containing 20 mM Tris (pH 7.5), 120 mM NaCl, 0.5% NP-40, 100 mM NaF, 0.25% Na-deoxycholate, 1 mM EDTA, 10% glycerol, 1 mM Na_3VO_4, 2 mM PMSF and protease inhibitor cocktail. Proteins from kidney samples were extracted following manufacturer´s instructions (#TR118, Molecular Research Center, Inc). Protein concentrations were determined using a DC protein assay kit (Bio-Rad). 20 µg of proteins were treated for 30 min at 50°C in a loading buffer containing 2% SDS and 5% β-mercaptoethanol (α-synuclein detection) or for 5 min at 95°C (other investigated proteins). The samples were electrophoresed on 8%, 10%, 12% or 15% SDS-PAGE gels, as appropriate, and transferred to PVDF membrane (pore size 0.45 µm, Immobilon-P, Millipore). For the detection of α-synuclein, post-transfer membranes were treated with 0.4% paraformaldehyde/PBS for 30 min at RT[3]. Membranes were blocked for 1 hour with 5% skim milk in Tris-buffer saline solution containing 0.1% Tween-20 (TBST) and subsequently probed with primary antibody against α-synuclein (#610786, BD Biosciences; 1/1000), β-synuclein (ab6165, Abcam; 1/1000), γ-synuclein (sc-65979; Santa Cruz; 1/800), E-cadherin (#610181, BD Biosciences; 1/2500), α-SMA (A5228, Sigma; 1/24000), Vimentin (#550513, BD Pharmingen; 1/17000), collagen I (COL1A1, AB765P, Chemicon; 1/2000), fibronectin (AB2033, Chemicon; 1/1000), α-tubulin (#T5168, Sigma;

1/5000), GAPDH (#919501, BioLegend), phospho-specific Akt (Ser 473) (#4060S, Cell Signaling; 1/2000), phospho-specific Erk1/2 (Thr 202/Tyr 204) (#675502, BioLegend; 1/5000), phospho-specific p-38 (Tyr 182) (E-1, sc-166182, Santa Cruz; 1/1000), phospho-specific MKK3 (Ser189)/MKK6 (Ser207) (#12280, Cell Signaling; 1/1000), total Erk1/2 (#686902, BioLegend; 1/1000), HA (3F10) (11867423001, Sigma; 1/1000) and Flag (11508721, Thermo Scientific; 1/1000) over night at 4°C. Horseradish peroxidase-conjugated secondary antibodies (anti-mouse, #115-035-003, Jackson Immunoresearch; anti-rabbit, #7074, Cell Signaling) were used at 1/10.000 for 1 hour, RT. The immunoreaction was visualized using chemiluminescent kits EZ ECL (Biological Industries) or ECL Advanced (Amersham Biosciences). Images were digitally acquired by ChemiDoc™ MP Imaging System (Bio-Rad). Positive immunoreactive bands were quantified by densitometry and compared with the expression of adequate loading control.

Immunofluorescence

For immunofluorescence analysis, cells were grown on coverslips in previously described conditions. After incubation, cells were washed twice with PBS and fixed with 4% Paraformaldehyde/PBS for 8 min. After permeabilization with 0.1% Triton X-100/PBS for 5 min, blocking of non-specific binding was performed by incubating cells with horse serum (Vector) for 30 minutes. Subsequently, cells were incubated with primary antibody for α-synuclein (#610786, BD Biosciences; 1/250), β-synuclein (ab6165, Abcam; 1/2000), γ-synuclein (sc-65979, Santa Cruz; 1/200) or p38 (#622401, BioLegend; 1/100) overnight at 4°C. After washing with PBS, cells were incubated with corresponding Alexa Fluor secondary antibody (Invitrogen) for 1 hour, RT. For F-actin labeling (actin filaments), cells were incubated with Alexa Fluor 568 Phalloidin (A12380, Invitrogen; 1/80) for 1 hour, RT.

Nuclear counterstaining was performed with Hoechst H33258 (Sigma) for 10 minutes, RT.

Paraformaldehyde-fixed mouse kidneys were immersed in 30% sucrose overnight and embedded in Bright Cryo-M-Bed compound for freezing. Slides were washed with PBS and incubated with Carbo Free Blocking solution (SP 5040, Vector) for 30 min. Blocking of non-specific binding was performed with 1% BSA/PBS for 30 min and sections were incubated with anti-rabbit α-synuclein (#4179S, Cell Signaling; 1/100) and LTA (B-1325, Vector Laboratories; 1/400) overnight at 4°C. After washing with PBS-Tween (0,05%), sections were incubated with anti-rabbit Alexa Fluor 488 secondary antibody (A11008, Invitrogen; 1/200) (visualization of α-synuclein) and Streptavidin Alexa Fluor 594 Conjugate (S32356, Invitrogen; 1/2000) (visualization of LTA) for one hour at RT. Slides were counterstained with Hoechst H33258. Sections were mounted with Fluoromount-G medium (0100-01, Southern Biotech). Stained cells and tissue sections were examined using an Olympus FluoView FV1000 confocal laser-scanning microscope with a Digital Camera System. For triple immunofluorescence staining of frozen mouse kidney sections, primary antibodies for E-cadherin (#147302, BioLegend; 1/200) or vimentin (#550513, BD Pharmingen; 1/400) were detected with anti-rat Dylight 649 (#712-496-153, Jackson Immunoresearch; 1/500) or anti-mouse Alexa Fluor 546 (A11003, Invitrogen; 1/200) secondary antibodies, respectively.

Morphometric analysis of interstitial fibrosis and immunohistochemistry

For histological examination, paraffin-embedded kidney sections were stained with Masson-Trichrome and Sirius Red for interstitial collagen content. After deparaffinization and hydratation, sections of kidneys were incubated in Picrosirius red solution (1% Sirius red in saturated picric acid) for 30 minutes at RT. This was followed by dehydratation in absolute ethanol. Quantification of collagen content after Sirius Red staining was made by determining the % of staining area in 4 randomly chosen fields (x40) using Image-Pro Plus Software. Data are expressed as positive stained area vs. total analyzed area. All samples were examined in a blind manner.

Immunostaining for α-synuclein, α-SMA and FSP1 was carried out on 5 μm thick tissue sections that were deparaffinized through xylene and rehydrated through graded ethanol concentrations (100%, 95% and 75%) and distilled water. Antigen retrieval was done in 10 mM citrate buffer (pH6) for 10 minutes. Endogenous peroxidase quenching (30 minutes incubation in 0.3% (v/v) H_2O_2/PBS)) was followed by blocking of non-specific binding for 1 hour at RT with 3% BSA/PBS. Primary antibodies for the detection of mouse α-synuclein (#4179S, Cell Signaling) or human α-synuclein (#610786, BD Biosciences), α-SMA (A5228, Sigma) and FSP1/S100A4 (ab27957, Abcam) were incubated overnight at 4ºC. After washing in PBS, slides were treated with the corresponding anti-IgG biotinylated secondary antibody followed by the avidin-biotin-peroxidase complex and 3,3'-diaminobenzidine as chromogen (all from Vector Laboratories, Inc. USA). Sections were counterstained with hematoxylin. Negative controls were performed by incubation with non-specific immunoglobulin of the same isotype as the primary antibody but with the omission of primary antibody. After immunostaining, slides were dehydrated, cleared in xylene and mounted with DPX permanent mounting medium. Stained tissue sections were

examined using an Olympus BX50 microscope with an Olympus automatic camera system. Immunohistochemical results of SNCA and α-SMA staining in kidney samples of CKD patients were evaluated following the uniform pre-established criteria. Staining intensity and % of positive cells were graded semiquantitatively. Histological scores were obtained from each sample as follows: histoscore = 1X (% light staining) + 2X (% moderate staining) + 3X (% strong staining), which ranged from 0 (no immunoreaction) to 300 (maximum immunoreactivity). The reliability of such scores for interpretation of immunohistochemical staining of tissue sections has already been reported[4].

SUPPLEMENTARY FIGURES

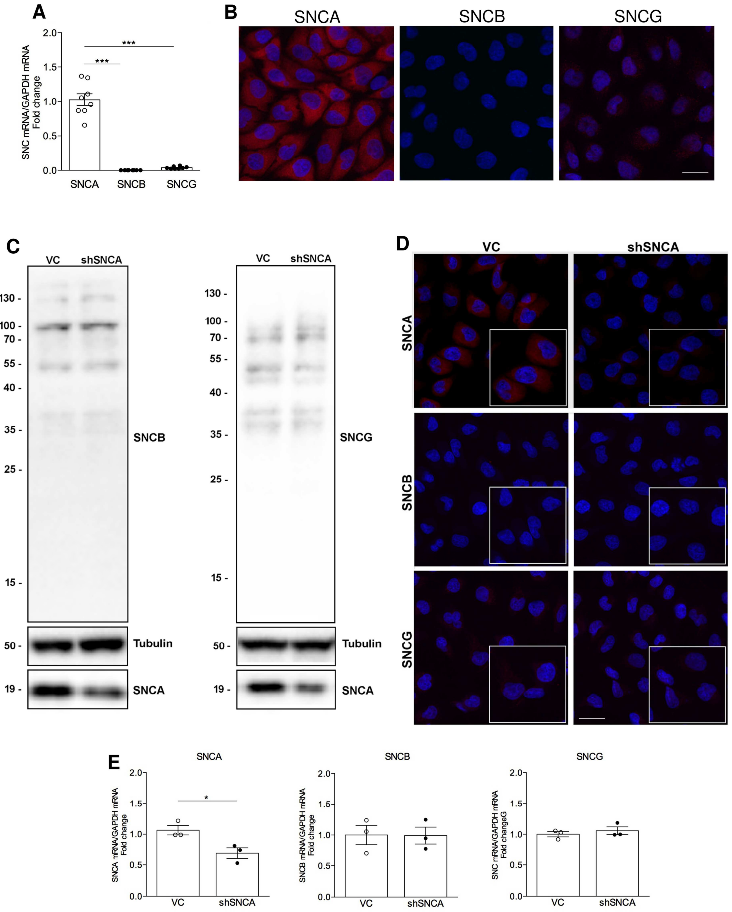

Supplementary Figure 1. Presence of α-synuclein (SNCA), β-synuclein (SNCB) and γ-synuclein (SNCG) in human renal proximal tubular epithelial (HK-2) cells. (**A**) Total mRNA was extracted from HK-2 cells (basal state) and mRNA levels of SNCA, SNCB and SNCG were determined by quantitative real-time PCR and normalized to GAPDH. Data are presented as mean ± SEM of three independent experiments assayed in triplicate for every condition. (**B, D**) Immunofluorescence staining for the presence of SNCA, SNCB and SNCG in HK-2 cells in basal state (**B**) and after downregulation of SNCA (VC vs. shSNCA) (**D**). Scale bar represents 20 µm. (**C**) HK-2 (VC and shSNCA) cell lysates were immunoblotted with antibodies against SNCA, SNCB and SNCG. The same samples were reprobed with antibodies against tubulin to ensure equal loading. Representative Western blots (**C**) show expression of SNCA, SNCB and SNCG in VC and shSNCA cells. Monomers of SNCB and SNCG were not detected neither in control nor in shSNCA HK-2 cells. Furthermore, no differences were found in oligomeric SNCB and SNCG in HK-2 with SNCA deficiency compared with control cells. (**E**) mRNA levels of SNCA, SNCB and SNCG in HK-2 cells (VC vs. shSNCA) were determined by quantitative real-time PCR after normalizing to GAPDH. Data are presented as mean ± SEM. *p<0,05 (Student´s t-test), ***p<0,001 (1-way ANOVA). VC – control vector; shSNCA – cells with SNCA downregulation. Source data are provided as a Source Data file.

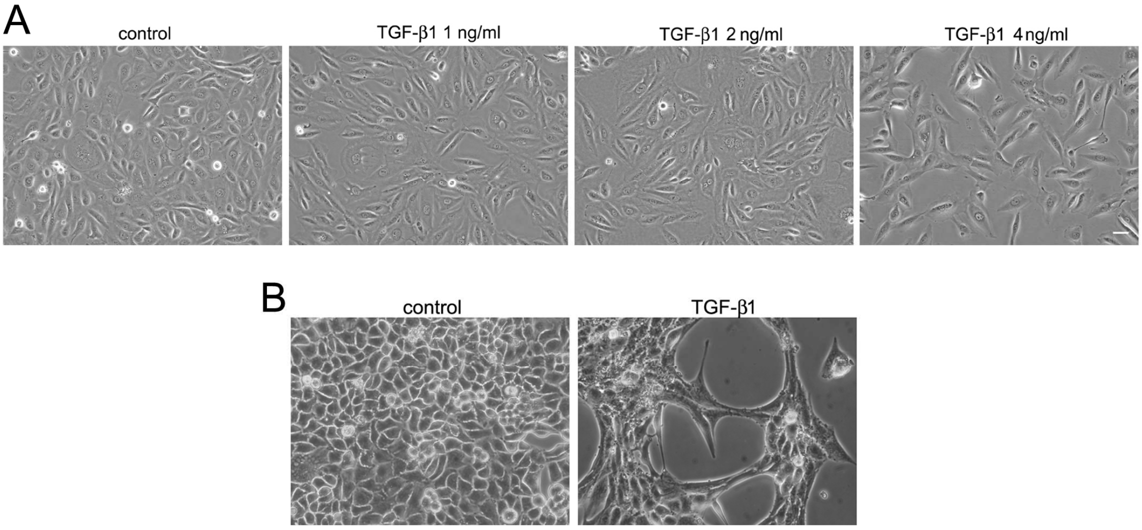

Supplementary Figure 2. Morphological changes in HK-2 cells upon treatment with TGF-β1. HK-2 cells were incubated in serum-free medium (control) or treated with TGF-β1 (1, 2, 4 ng/ml) for different periods of time. (**A, B**) Treatment of HK-2 cells with TGF-β1 induced visible changes in cell morphology toward the loss of cobble-stoned shape and acquisition of spindle-like form of the cell (**A**, 24h; **B**, 72h). Scale bar represents 10 µm.

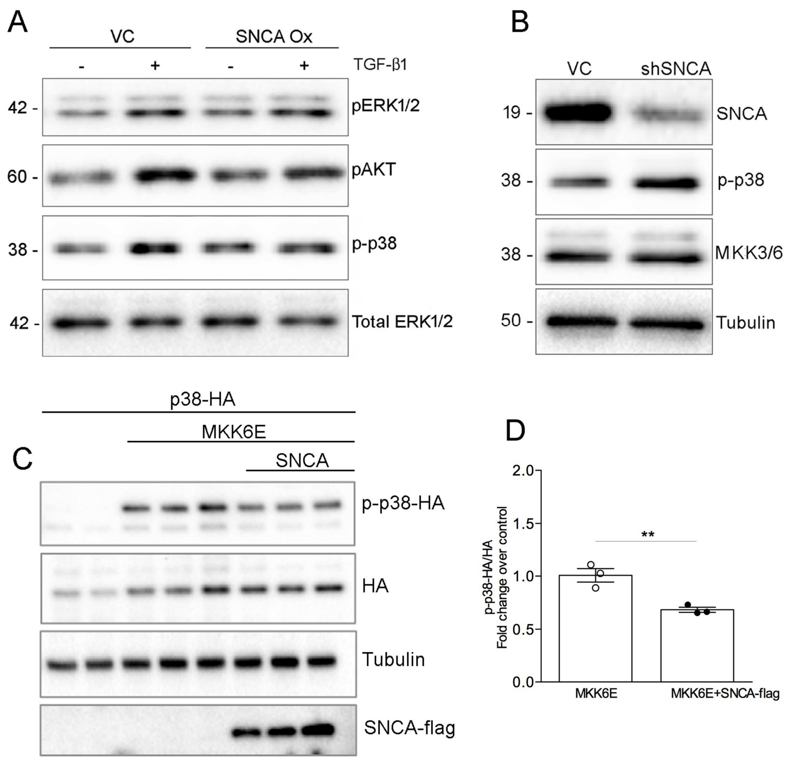

Supplementary Figure 3. Effect of SNCA on the activity of MAPK and PI3K pathways *in vitro*. (**A**) HK-2 cells were incubated separately with either serum-free medium or 2 ng/ml TGF-β1. Cell lysates were immunoblotted with antibodies against pERK1/2, pAkt, p-p38 and total ERK1/2. Representative Western blots show levels of phosphorylated ERK1/2, Akt and p38 in HK-2 cells. (**B**) Knockdown of SNCA in HK-2 cells does not change the expression of pMKK3/6. Representative Western blots show levels of SNCA, p-p38 and pMKK3/6 in HK-2 cells. (**C**, **D**) HEK293T cells were co-transfected with SNCA-flag, p38-HA and MKK6E plasmids. Cell lysates were immunoblotted with antibodies against p-p38, flag and HA. Representative Western blots (**C**) and quantitative densitometric analysis (**D**) show that overexpression of SNCA managed to decrease the levels of phosphorylated p38 in cells with constitutive activation of MKK6 kinase. Data are presented as mean ± SEM. **p<0,01 by Student´s t-test. shSNCA – cells with SNCA downregulation; SNCA Ox – cells overexpressing SNCA. Source data are provided as a Source Data file.

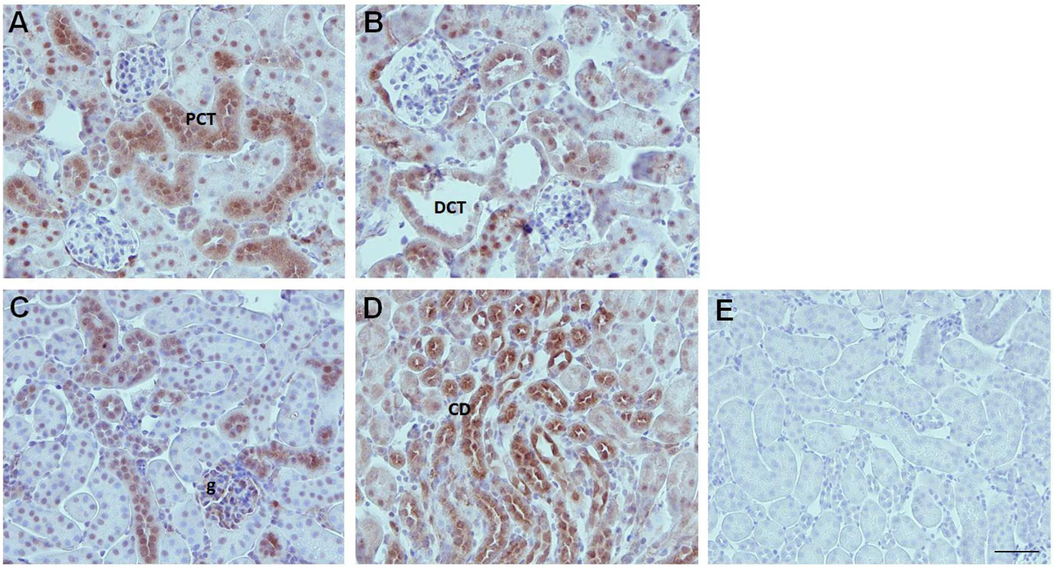

Supplementary Figure 4. Localization of SNCA in normal mouse kidney. Representative micrographs of immunoperoxidase staining illustrating the presence SNCA in mouse kidney. PCT; proximal convoluted tubule, DCT, distal convoluted tubule; g, glomerulus; CD, collecting duct. (E) kidney section representing negative control. Scale bar represents 50 µm.

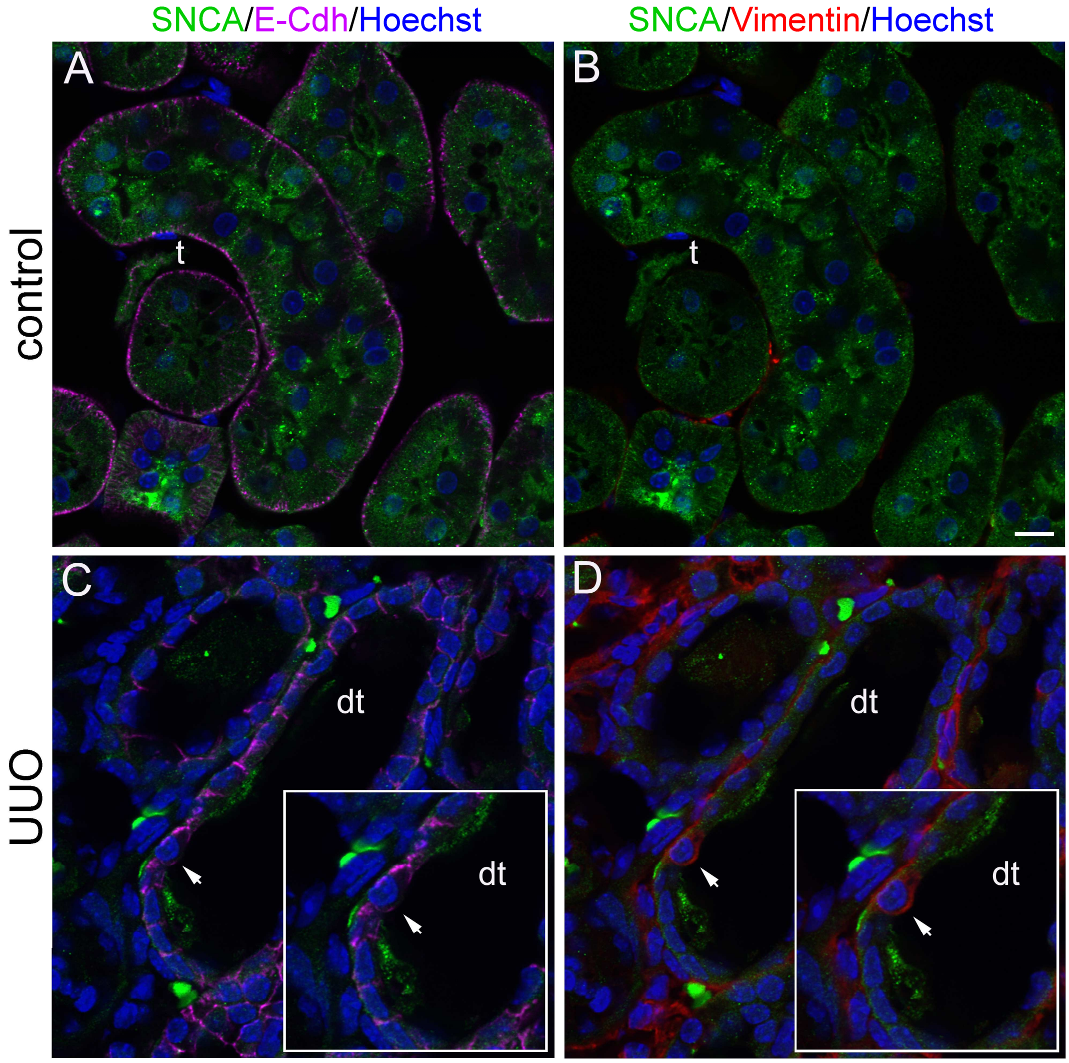

Supplementary Figure 5. Triple immunofluorescence staining for SNCA, E-cadherin and vimentin in mouse kidney after UUO-induced renal fibrosis. PEPCK^{Cre+} SNCA$^{wt/wt}$ mice were subjected to UUO and kidneys were collected 15 days after surgery. Representative images of kidney sections from contralateral non-obstructed (control) (**A**, **B**) and obstructed (UUO) kidneys (**C**, **D**) stained for SNCA, E-cadherin and vimentin and counterstained for Hoechst to visualize nuclei. The selected field (white rectangle) in the photomicrograph of the UUO kidney (**C**, **D**) illustrates a tubular cell in the area of fibrosis (arrow) that lost SNCA expression and gained *de novo* vimentin expression (**D**), but still preserving some levels of E-cadherin expression (**C**). Scale bar represents 10 µm. t, tubule; dt, dilated tubule.

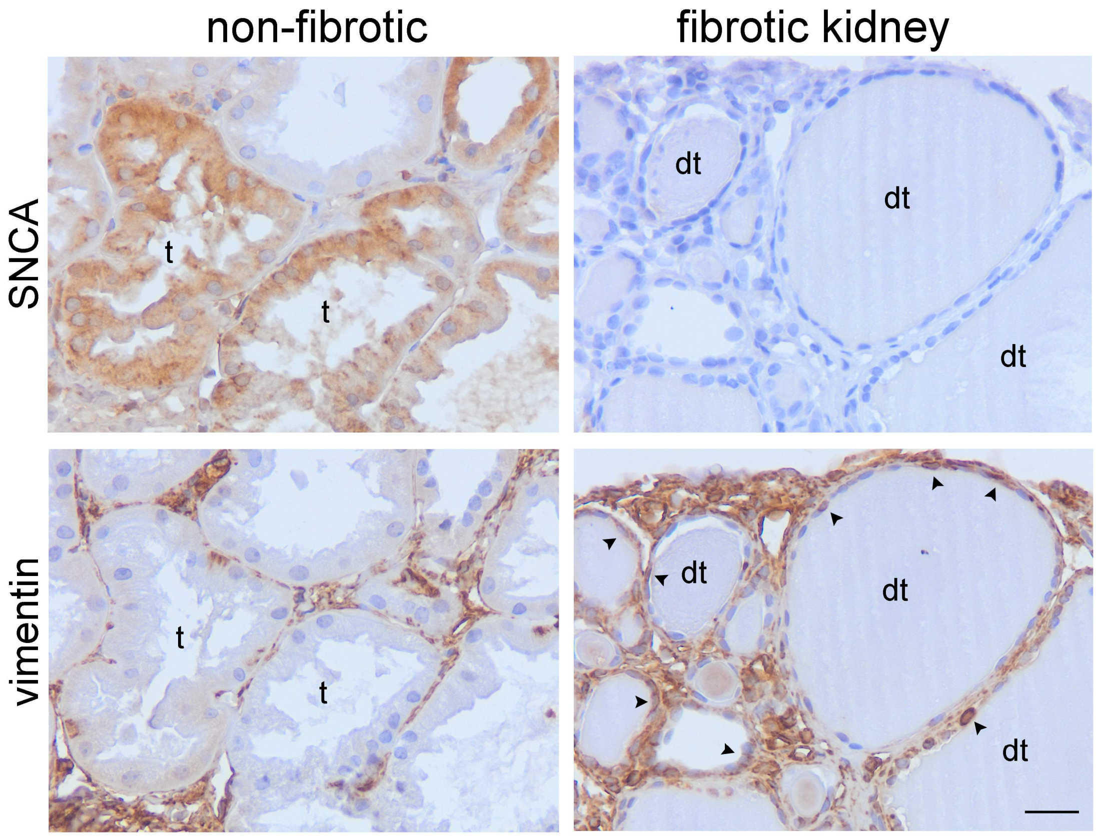

Supplementary Figure 6. Immunoperoxidase staining for SNCA and vimentin in human kidney affected by fibrosis. Representative images of immunoperoxidase staining for SNCA and vimentin in serial sections of human kidney. Black arrows in the photomicrograph of the fibrotic kidney point to tubular cells in the area of fibrosis that lost SNCA expression and gained *de novo* vimentin expression (D). Scale bar represents 20µm. t, tubule; dt, dilated tubule.

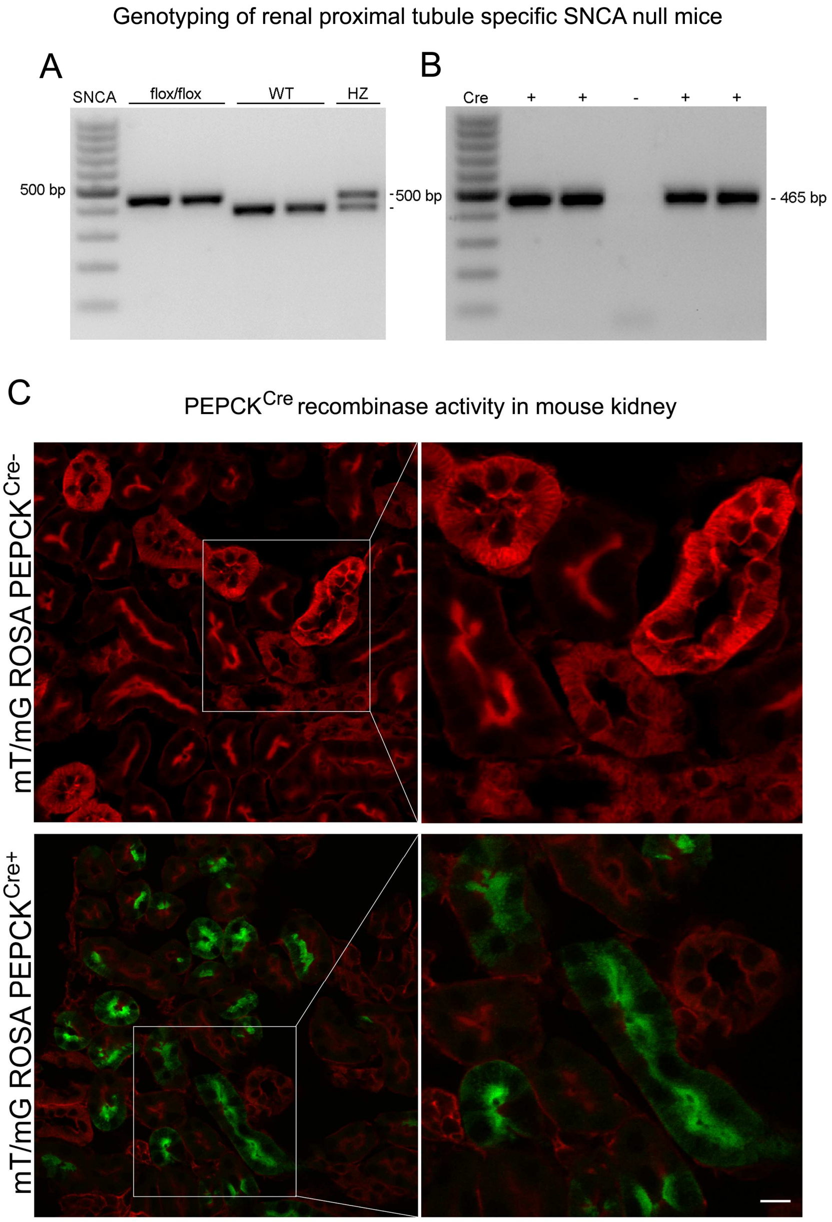

Supplementary Figure 7. Generation and confirmation of RPTEC-specific SNCA knockout mice. (**A**) PCR genotyping of wild type (WT) and floxed SNCA gene alleles using primers flanking exon 2 of the SNCA gene. WT SNCA alleles produced a 390 bp product while the floxed allele produced a 440 bp product. (**B**) Mice positive for the Cre transgene (+) showed a PCR product of an approximately 465 bp. (**C**) Kidney EGFP reporter expression characteristic of Cre recombinase activity (green fluorescence) in B6.129(Cg)-Gt(ROSA)26Sor$^{tm4(ACTB\text{-}tdTomato\text{-}EGFP)Luo/J}$ mouse (red fluorescence) crossed with PEPCK^{Cre+} mouse. Scale bar represents 10 µm. SNCA, α-synuclein; RPTEC – renal proximal tubular epithelial cell.

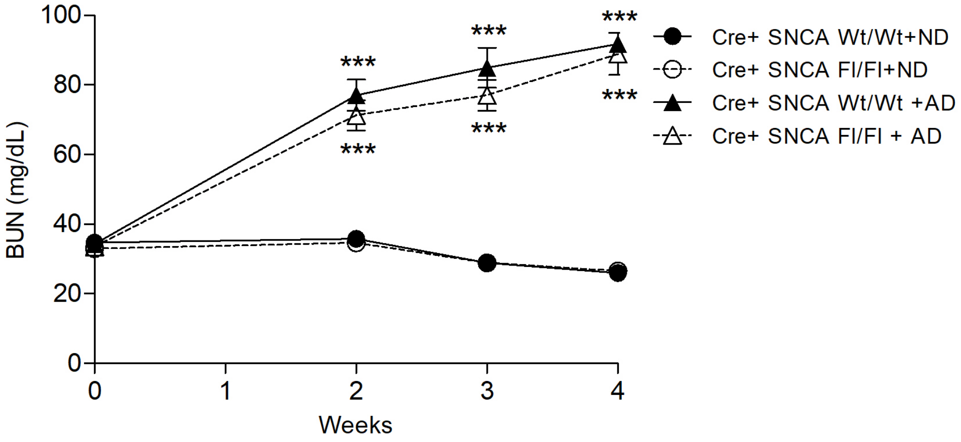

Supplementary Figure 8. Adenine diet feeding induced progressive decline of renal function in PEPCK^{Cre+} SNCA$^{wt/wt}$ and PEPCK^{Cre+} SNCA$^{fl/fl}$ mice. PEPCK^{Cre+} SNCA$^{wt/wt}$ and PEPCK^{Cre+} SNCA$^{fl/fl}$ mice were fed an adenine-rich diet or standard (control) diet during 4 weeks. Renal function was assessed by measuring serum blood urea nitrogen (BUN) (mg/dL). Data are presented as mean ± SEM (n=5-7 mice/group). ***p<0,001 (two-way ANOVA) vs. PEPCK^{Cre+} SNCA$^{wt/wt}$ standard diet or PEPCK^{Cre+} SNCA$^{fl/fl}$. Source data are provided as a Source Data file.

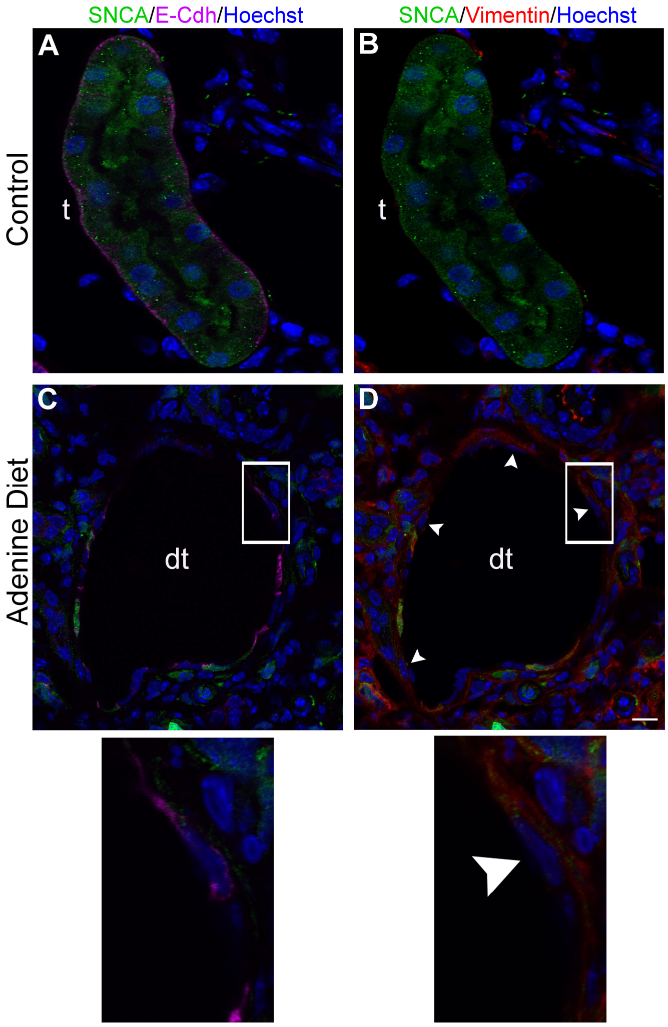

Supplementary Figure 9. Triple immunofluorescence staining for SNCA, E-cadherin and vimentin in mouse kidney after adenine-induced renal fibrosis. PEPCK^{Cre+} SNCA$^{wt/wt}$ mice were fed a regular or adenine rich diet and kidneys were collected 4 weeks after the commencement of the experiment. Representative images of kidney sections from control mice fed a regular diet (**A**, **B**) and mice fed an adenine diet (**C**, **D**) stained for SNCA, E-cadherin and vimentin and counterstained for Hoechst to visualize nuclei. Selected fields (white rectangles) in the photomicrograph (**C, D**) illustrates a tubular cell in the area of fibrosis (arrow) that lost SNCA expression and gained *de novo* vimentin expression (**D**), but still preserving some levels of E-cadherin expression (**C**). Other arrowheads on the panel **D** indicate various tubular cells, with decreased or lost expression of SNCA, showing *de novo* vimentin expression. Scale bar represents 10 µm. t, tubule; dt, dilated tubule.

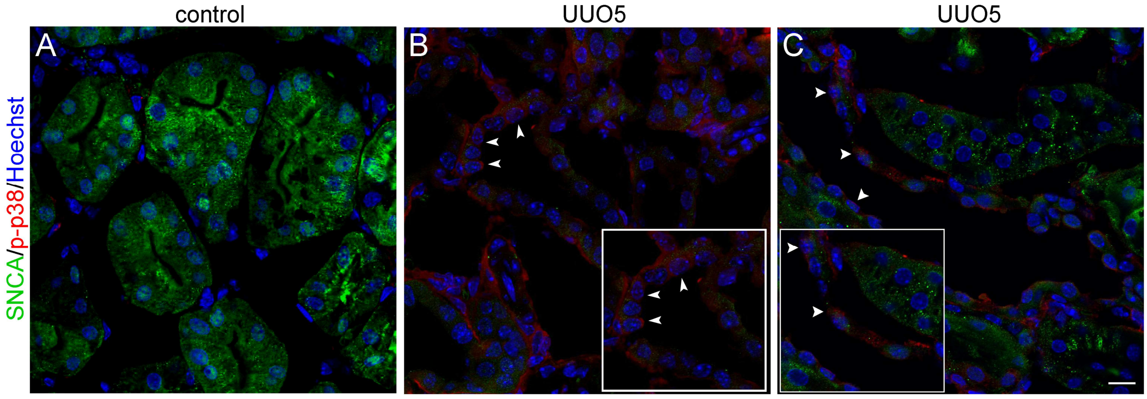

Supplementary Figure 10. Increase of p-p38 in mouse tubular cells lacking SNCA expression. PEPCK^{Cre+} SNCA$^{wt/wt}$ mice were subjected to UUO and kidneys were collected 5 days after surgery. Representative images from kidney sections from contralateral non-obstructed (control) (**A**) and obstructed (UUO5) (**B, C**) kidneys stained for SNCA and p-p38 and counterstained for Hoechst to visualize nuclei. The selected field (white rectangle) in the photomicrograph of the UUO kidneys (**B, C**) illustrates tubular cells in the area of fibrosis (arrows) that showed decreased or lost SNCA expression and gained p-p38 expression. Scale bar represents 10 µm.

Table S1. Characteristics of patients

Number of patients	43		
Age (years)	59.02 ± 16.9		
Gender (male, N, %)	20 (46.5%)		
BMI (kg/m²)	27.3 ± 6.7		
Systolic BP (mmHg)	132.5 ± 20.4		
Diastolic BP (mmHg)	74.9 ± 10.9		
Serum albumin (g/dL)	3.7 ± 0.9		
Proteinuria (mg/L)	787.1 ± 912.7		
eGFR (mL/min/1.73 m²)	54.5 ± 24.4		
Medical diagnosis	N (%)	eGFR	Proteinuria
Cancer	14 (32.6%)	52.0 ± 15.6	239.0 ± 482.2
IgA nephropathy (Berger´s disease)	6 (13.93%)	49.3 ± 34.1	661.05 ± 249.1
CP/Hydronephrosis	5 (11.62%)	46.6 ± 22.3	10.7 ± 12.7
Tubulointerstitial nephritis	4 (9.3%)	58.7 ± 33.1	800.2 ± 1193.4
Nephroangiosclerosis	4 (9.3%)	51.9 ± 5.6	661.05 ± 249.1
Glomerulonephritis	3 (6.98%)	58.0 ± 44.9	1397.8 ± 1225.7
Other KDs	7 (16.27%)	65.3 ± 23.5	1414.2 ± 1064.5

Supplementary Table 1. Characteristics of patients. Data are shown as mean ± standard deviation or as number of cases (%). BMI, body mass index; BP, blood pressure; eGFR, estimated glomerular filtration rate; CP, chronic pyelonephritis; Other KDs, other kidney diseases (Focal segmental glomerulosclerosis (n=2), Diabetic nephropathy (n=2), Membranous nephropathy (n=2), Minimal change disease (n=1)).

References

1. Tamura, M, Aizawa, R, Hori, M, Ozaki, H: Progressive renal dysfunction and macrophage infiltration in interstitial fibrosis in an adenine-induced tubulointerstitial nephritis mouse model. *Histochem Cell Biol,* 131**:** 483-490, 2009.
2. Jia, T, Olauson, H, Lindberg, K, Amin, R, Edvardsson, K, Lindholm, B, Andersson, G, Wernerson, A, Sabbagh, Y, Schiavi, S, Larsson, TE: A novel model of adenine-induced tubulointerstitial nephropathy in mice. *BMC Nephrol,* 14**:** 116, 2013.
3. Lee, BR, Kamitani, T: Improved immunodetection of endogenous α-synuclein. *PLoS One,* 6**:** e23939, 2011.
4. Torremade, N, Bozic, M, Panizo, S, Barrio-Vazquez, S, Fernandez-Martin, JL, Encinas, M, Goltzman, D, Arcidiacono, MV, Fernandez, E, Valdivielso, JM: Vascular Calcification Induced by Chronic Kidney Disease Is Mediated by an Increase of 1 alpha-Hydroxylase Expression in Vascular Smooth Muscle Cells. *Journal of Bone and Mineral Research,* 31**:** 1865-1876, 2016.
