# Supplementary material for: Protective role of renal proximal tubular alpha-synuclein in the pathogenesis of kidney fibrosis
Source: Nat Commun. 2020 Apr 23;11:1943. doi: 10.1038/s41467-020-15732-9 (PMC7181766; doi:10.1038/s41467-020-15732-9)
Supplement: Supplementary file 3 — Reporting Summary [file 41467_2020_15732_MOESM3_ESM.pdf]

nature research

Corresponding author(s): Milica Bozic and Jose M. Valdivielso

Last updated by author(s): Jan 29, 2020

Reporting Summary

Nature Research wishes to improve the reproducibility of the work that we publish. This form provides structure for consistency and transparency in reporting. For further information on Nature Research policies, see Authors & Referees and the Editorial Policy Checklist .

Statistics

For all statistical analyses, confirm that the following items are present in the figure legend, table legend, main text, or Methods section.

n/a	Confirmed	
☐	☒	The exact sample size (*n*) for each experimental group/condition, given as a discrete number and unit of measurement
☐	☒	A statement on whether measurements were taken from distinct samples or whether the same sample was measured repeatedly
☐	☒	The statistical test(s) used AND whether they are one- or two-sided<br>*Only common tests should be described solely by name; describe more complex techniques in the Methods section.*
☒	☐	A description of all covariates tested
☒	☐	A description of any assumptions or corrections, such as tests of normality and adjustment for multiple comparisons
☐	☒	A full description of the statistical parameters including central tendency (e.g. means) or other basic estimates (e.g. regression coefficient) AND variation (e.g. standard deviation) or associated estimates of uncertainty (e.g. confidence intervals)
☐	☒	For null hypothesis testing, the test statistic (e.g. *F*, *t*, *r*) with confidence intervals, effect sizes, degrees of freedom and *P* value noted<br>*Give P values as exact values whenever suitable.*
☒	☐	For Bayesian analysis, information on the choice of priors and Markov chain Monte Carlo settings
☒	☐	For hierarchical and complex designs, identification of the appropriate level for tests and full reporting of outcomes
☒	☐	Estimates of effect sizes (e.g. Cohen's *d*, Pearson's *r*), indicating how they were calculated

Our web collection on statistics for biologists contains articles on many of the points above.

Software and code

Policy information about availability of computer code

Data collection	BioRad CFX Manager Software version 3.1; Image Lab Software version 4.0.1; Olympus cell Sens Entry version 1.16; Olympus FV10-ASW Viewer version 4.1.
Data analysis	BioRad CFX Manager Software ver 3.1; Image Lab Software version 4.0.1; GraphPad Prism ver 5.01.

For manuscripts utilizing custom algorithms or software that are central to the research but not yet described in published literature, software must be made available to editors/reviewers. We strongly encourage code deposition in a community repository (e.g. GitHub). See the Nature Research guidelines for submitting code & software for further information.

Data

Policy information about availability of data

All manuscripts must include a data availability statement. This statement should provide the following information, where applicable:

- Accession codes, unique identifiers, or web links for publicly available datasets
- A list of figures that have associated raw data
- A description of any restrictions on data availability

Source data are provided as a Source Data file. All other data supporting the findings of this study are included in the supplementary information or available from the corresponding author upon reasonable request.

Field-specific reporting

Please select the one below that is the best fit for your research. If you are not sure, read the appropriate sections before making your selection.

Life sciences study design

All studies must disclose on these points even when the disclosure is negative.

Sample size	No statistical methods were used to predetermine the sample size. Sample size was determined to be adequate based on previous reports, and in the consistency of measurable differences between groups. Sample size of biopsies from human kidneys was 43, as it was the maximum number of biopsies with informed consent found in the Biobank.The precise number of mice used in in vivo study, as well as the number of replicates in in vitro study is given in the corresponding figure legend.
Data exclusions	For in vitro experiments, data were only excluded for failed experiments. For in vivo experiments, if unilateral obstruction surgery was unsuccessful, the animal was excluded from the study.
Replication	Experiments were repeated successfully. The number of repeats are given in the corresponding figure legend.
Randomization	We did not use randomization to allocate mice to experimental groups as the design allows each mice to be its own control. Mice analyzed were litter mates and male gender. Information on mouse groups is included in the Methods section.
Blinding	Investigators were not blinded to mouse genotypes and group allocation during experiments. Analysis of stainings in human and mice samples was blinded.

Reporting for specific materials, systems and methods

We require information from authors about some types of materials, experimental systems and methods used in many studies. Here, indicate whether each material, system or method listed is relevant to your study. If you are not sure if a list item applies to your research, read the appropriate section before selecting a response.

Materials & experimental systems

n/a	Involved in the study	
☐	☒	Antibodies
☐	☒	Eukaryotic cell lines
☒	☐	Palaeontology
☐	☒	Animals and other organisms
☐	☒	Human research participants
☐	☒	Clinical data

Methods

n/a	Involved in the study	
☒	☐	ChIP-seq
☒	☐	Flow cytometry
☒	☐	MRI-based neuroimaging

Antibodies

Antibodies used	α-synuclein (#610786, BD Biosciences); E-cadherin (#610181, BD Biosciences); α-SMA (A5228, Sigma); Vimentin (#550513, BD Pharmingen); collagen I (COL1A1, AB765P, Chemicon); fibronectin (AB2033, Chemicon); α-tubulin (#T5168, Sigma); GAPDH (#919501, BioLegend); phospho-specific Akt (Ser 473) (#4060S, Cell Signaling); phospho-specific Erk1/2 (Thr 202/Tyr 204) (#675502, BioLegend); phospho-specific p-38 (Tyr 182) (E-1, sc-166182, Santa Cruz); phospho-specific MMK3 (Ser189)/MMK6 (Ser207) (#12280, Cell Signaling); total Erk1/2 (#686902, BioLegend); anti-mouse, #115-035-003, Jackson Immunoresearch; anti-rabbit, #7074, Cell Signaling; β-synuclein (ab6165, Abcam); gamma-synuclein (sc-65979, Santa Cruz); p38 (#622401, BioLegend); α-synuclein (#4179S, Cell Signaling); Alexa Fluor 568 Phalloidin (A12380, Invitrogen); E-cadherin (#147302, BioLegend); vimentin (#550513, BD Pharmingen); anti-rat Dylight 649 (#712-496-153, Jackson Immunoresearch), anti-mouse Alexa Fluor 546 (A11003, Invitrogen); FSP1/S100A4 (ab27957, Abcam); HA (3F10) (11867423001, Sigma); Flag (11508721, Thermo Scientific).
Validation	All the antibodies are validated by the suppliers on their webpages.

Eukaryotic cell lines

Policy information about cell lines

Cell line source(s)	HK-2 and HEK293 cells were purchased from ATCC.
Authentication	Autentication was provided by supplier. However, HK2 cells have a special coblestone pattern when confluent that was used to confirm its authenticity.
Mycoplasma contamination	All cells were negative for Mycoplasma contamination.
Commonly misidentified lines (See ICLAC register)	No commonly misidentified cell lines were used.

Animals and other organisms

Policy information about studies involving animals; ARRIVE guidelines recommended for reporting animal research

Laboratory animals	We used male C57BL/6J mice; SNCAflox mice [B6(Cg)- Sncatm1.1Vlb/J; JAX stock #025636]; C57BL/6J PEPCKCre+ transgenic mice. Description of research mice used for experiments are explained in details in Methods section.
Wild animals	This study did not involve wild animals.
Field-collected samples	This study did not involve samples collected from the field.
Ethics oversight	Animal Ethics Committee of the University of Lleida (CEEA 07-02/14).

Note that full information on the approval of the study protocol must also be provided in the manuscript.

Human research participants

Policy information about studies involving human research participants

Population characteristics	Patients which underwent nephrectomy due to malignancies or hydronephrosis, and patients who were submitted to renal biopsy and were confirmed with IgA nephropathy, glomerulonephritis and chronic pyelonephritis.
Recruitment	Sequentially recruited at time of biopsy or nephrectomy. Human kidney samples were selected by two experienced pathologists who evaluated samples obtained from routine kidney biopsies for the presence of renal fibrosis. There are no biases that may impact the results.
Ethics oversight	Ethics Committee for the Clinical Investigation of the University Hospital Arnau de Vilanova in Lleida (CEIC-1587)

Note that full information on the approval of the study protocol must also be provided in the manuscript.

Clinical data

Policy information about clinical studies

All manuscripts should comply with the ICMJEguidelines for publication of clinical research and a completedCONSORT checklist must be included with all submissions.

Clinical trial registration	*Provide the trial registration number from ClinicalTrials.gov or an equivalent agency.*
Study protocol	*Note where the full trial protocol can be accessed OR if not available, explain why.*
Data collection	*Describe the settings and locales of data collection, noting the time periods of recruitment and data collection.*
Outcomes	*Describe how you pre-defined primary and secondary outcome measures and how you assessed these measures.*
